# Therapeutic Anabolic and Anticatabolic Benefits of Natural Chinese Medicines for the Treatment of Osteoporosis

**DOI:** 10.3389/fphar.2019.01344

**Published:** 2019-11-25

**Authors:** Jianbo He, Xiaojuan Li, Ziyi Wang, Samuel Bennett, Kai Chen, Zhifeng Xiao, Jiheng Zhan, Shudong Chen, Yu Hou, Junhao Chen, Shaofang Wang, Jiake Xu, Dingkun Lin

**Affiliations:** ^1^Guangzhou University of Chinese Medicine, Guangzhou, China; ^2^The School of Biomedical Sciences, University of Western Australia, Perth, WA, Australia; ^3^The Second Affiliated Hospital of Guangzhou University of Chinese Medicine, Guangdong Provincial Hospital of Chinese Medicine, Guangzhou, China; ^4^Formula-Pattern Research Center, School of Traditional Chinese Medicine, Jinan University, Guangzhou, China; ^5^Centre for Legumes in Mediterranean Agriculture, University of Western Australia, Perth, WA, Australia

**Keywords:** osteoporosis, natural Chinese medicine, osteogenesis, osteoclastogenesis, antibone resorption, therapeutic, osteoblasts, osteoclasts

## Abstract

Osteoporosis is a bone disease characterized by increasing osseous fragility and fracture due to the reduced bone mass and microstructural degradation. Primary pharmacological strategies for the treatment of osteoporosis, hormone replacement treatment (HRT), and alendronate therapies may produce adverse side-effects and may not be recommended for long-term usage. Some classic and bone-specific natural Chinese medicine are very popularly used to treat osteoporosis and bone fracture effectively in clinical with their potential value in bone growth and development, but with few adverse side-effects. Current evidence suggests that the treatments appear to improve bone metabolism and attenuate the osteoporotic imbalance between bone formation and bone resorption at a cellular level by promoting osteoblast activity and inhibiting the effects of osteoclasts. The valuable therapies might, therefore, provide an effective and safer alternative to primary pharmacological strategies. Therefore, the purpose of this article is to comprehensively review these classic and bone-specific drugs in natural Chinese medicines for the treatment of osteoporosis that had been deeply and definitely studied and reported with both bone formation and antiresorption effects, including *Gynochthodes officinalis* (F.C.How) Razafim. & B.Bremer (syn. *Morinda officinalis F.C.How*), *Curculigo orchioides Gaertn.*, *Psoralea corylifolia* (*L.*) *Medik Eucommia ulmoides Oliv.*, *Dipsacus inermis* Wall. (*syn. Dipsacus asperoides C.Y.Cheng & T.M.Ai*), *Cibotium barometz* (*L.*) *J. Sm.*, *Velvet Antler*, *Cistanche deserticola Ma*, *Cuscuta chinensis* Lam., *Cnidium monnieri* (L.) Cusson, *Epimedium brevicornum* Maxim, *Pueraria montana* (Lour.) Merr. and *Salvia miltiorrhiza* Bunge., thus providing evidence for the potential use of alternative Chinese medicine therapies to effectively treat osteoporosis.

## Introduction

Osteoporosis may result from imbalanced bone metabolism leading to a systemic deterioration in bone mass and bone microstructure, characterized by skeletal fragility and an increased risk of bone fracture (Albright, 1947; Rachner et al., 2011). Almost one in three women and one in five men would experience one bone fracture in their life after 50 years of age, resulting from osteoporosis (Sozen et al., 2017). Furthermore, the risk of additional fractures will rise exponentially with each incidence of fracture (Lorentzon and Cummings, 2015). Osteoporosis is, therefore, a debilitating disease for sufferers, leading to reduced quality of life, and places a large economic burden on society (Curtis et al., 2016). Thus, medical intervention is imperative to provide adequate care for patients and improve societal health. Moreover, it is necessary to continue research leading to the development of a medical treatment that may effectively treat and potentially prevent osteoporosis (Wang et al., 2017b).

Currently, there are numerous pharmacological products used for the treatment of osteoporosis in the clinic (Pavone et al., 2017). Hormone replacement therapy (HRT) and bisphosphonates are the primary therapeutic strategies for bone loss diseases including osteoporosis (He et al., 2017). Long-term HRT may significantly increase the risk of endometrial and mammary cancer, and coronary heart disease and other cardiovascular diseases (Dinger et al., 2016a; Dinger et al., 2016b). While bisphosphonates may lead to osteonecrosis of the long bones and jaws (Spivakovsky, 2017; Lungu et al., 2018). These adverse side-effects limit the clinical use of HRT and bisphosphonates. Therefore, alternative therapeutic agents are required to develop medicines for the treatment of osteoporosis that are less likely to have adverse side-effects.

**Figure 1:**
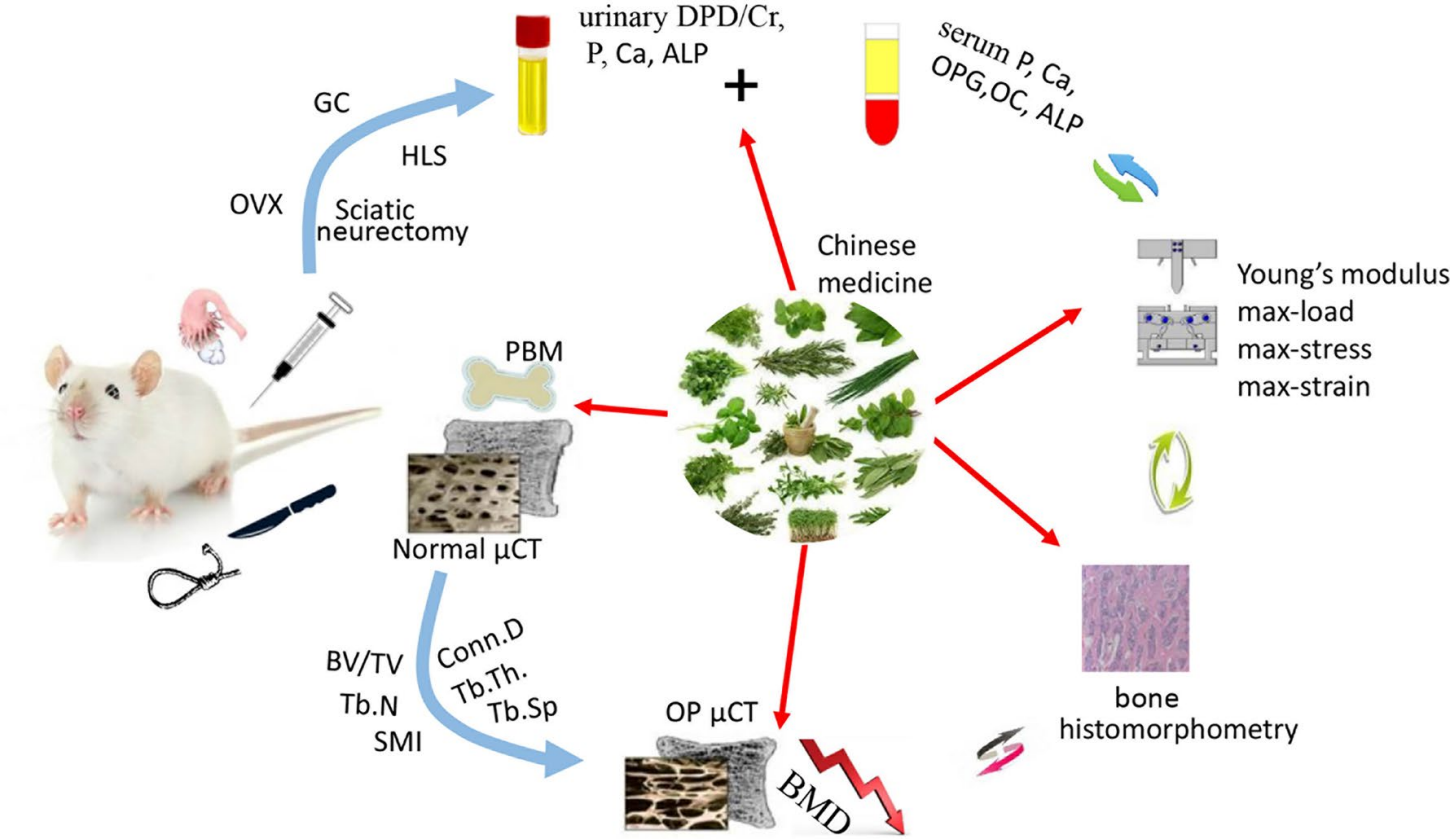
The therapeutic potential of natural Chinese medicine for the treatment of osteoporosis. The bone quality will be seriously impaired facing the challenges of estrogen or androgen deficiency, excessive hormone drugs, and weightlessness. While some of the natural Chinese medicines could act as potential candidates to improve the skeleton formation and inhibit bone loss. (OVX, ovariectomization; GC, Glucocorticoid; HLS, Hind Limb Suspension).

**Figure 2:**
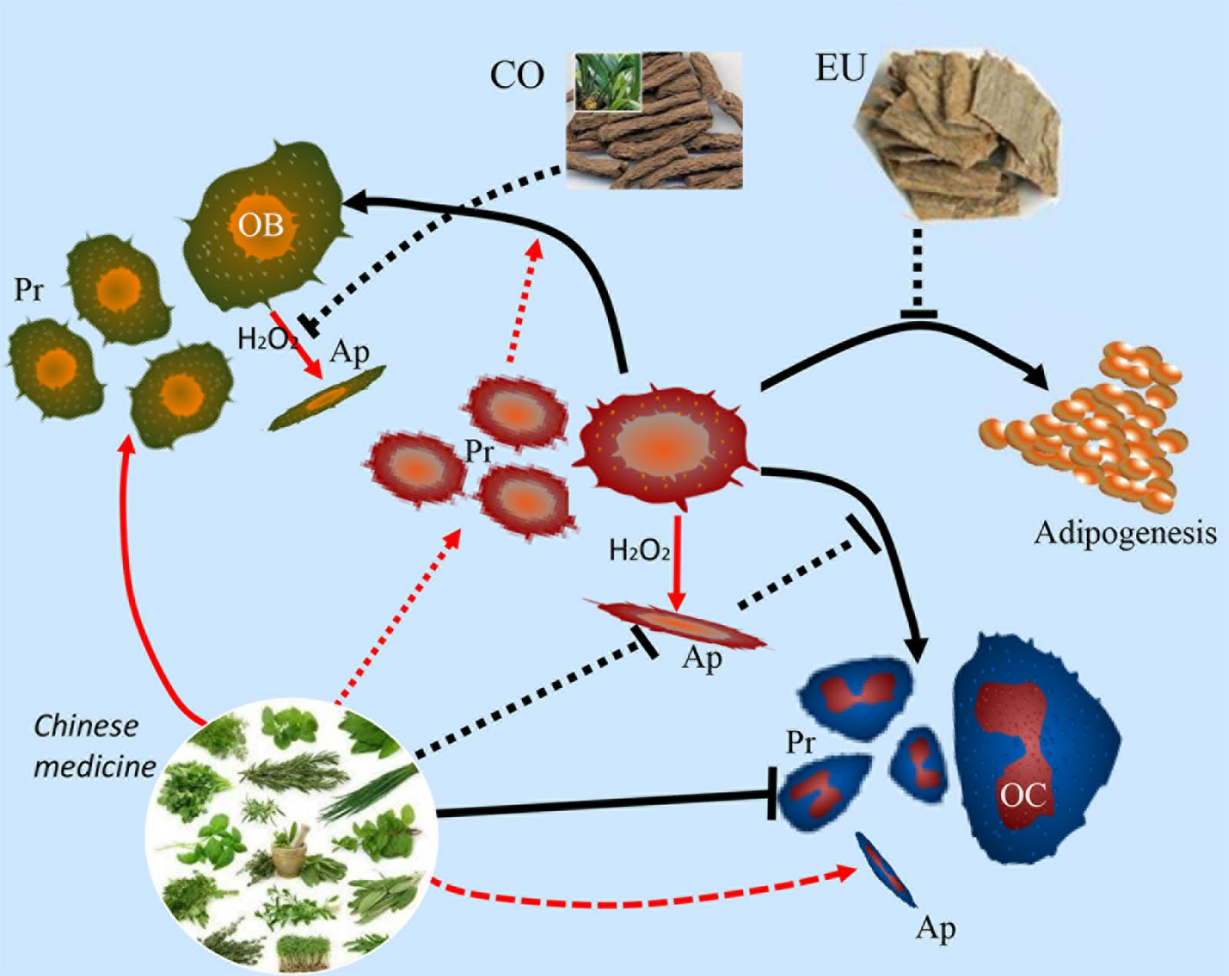
Natural Chinese medicine could promote the proliferation (Pr) and differentiation of osteoblasts and bone mesenchymal stem cells, enhance the osteogenesis ability, and inhibit the apoptosis (Ap) of osteoblasts induced by oxidative stress. While the osteoclastogenesis and bone-resorption function of osteoclasts are inhibited by their beneficial effects.

Traditional Chinese medicine (TCM) has become increasingly popular due to its effectiveness in treating diseases, with fewer side-effects. Natural Chinese medicine has been widely and effectively used to treat a variety of orthopaedic diseases, including bone fractures, rheumatism, and osteoporosis (Mukwaya et al., 2014; He et al., 2017; Suvarna et al., 2018). Some TCMs are the most classical and bone-specific of drugs when applied to the treatment of bone loss and bone fracture diseases, with the effects on the growth and development of skeleton tissue (Shu et al., 2015; Wang et al., 2019a). Recent scientific reports suggest that these natural Chinese medicine therapies appear to have both the anabolic and anticatabolic effects for the treatment of osteoporosis by promoting bone formation and attenuating imbalanced bone resorption, leading to improved bone mineral density and biomechanical properties, and reduced bone microstructural degradation (**Figure 1**) (He et al., 2017; Wang et al., 2017b; Suvarna et al., 2018). Further, *in vitro* findings indicate that these natural medicines may enhance the proliferation and survival of osteoblasts, and they could induce the differentiation of osteoblast cells from bone mesenchymal stem cells (MSCs). While the bone catabolic effects of osteoclastogenesis and bone resorption were effectively inhibited (**Figure 2**).

Therefore, we selected the natural Chinese medicines, which are the classical bone-specific drugs for the bone loss and fracture diseases in clinic, including *Gynochthodes officinalis* (F.C.How) Razafim. & B.Bremer (syn. *Morinda officinalis* F.C.How), *Curculigo orchioides* Gaertn., *Psoralea corylifolia (L.) Medik, Eucommia ulmoides* Oliv., *Dipsacus inermis* Wall. (syn. *Dipsacus asperoides C.Y.Cheng & T.M.Ai*), *Cibotium barometz* (L.) J. Sm., Velvet Antler, and so on. Then, according to the literature search of PubMed, each natural Chinese medicine was searched with the keywords of bone, osteoporosis, bone loss, osteolysis, bone formation, skeleton, osteogenesis, osteoclastogenesis, antibone resorption, bone resorption, bone absorption, therapeutic, osteoblasts, osteoclasts, bone mesenchymal stem cell, differentiation, apoptosis, formula, and combination, accompanied with the Boolean connectors of “AND”, “OR”, and “NOT”. After careful reading, the medicines with both anabolic and anticatabolic effects were included and reviewed. Medicines without osteoporosis-related research, or without both anabolic and anticatabolic benefits, or with flaws in experimental design were excluded. Despite the massive amount of experimental data regarding antiosteoporotic effects by the natural Chinese medicines from the *in vivo* and *in vitro* studies, high-quality clinical trials are lacking. Additionally, this review highlights and evaluates the scientific evidence for the potential use of natural Chinese medicines in the treatment of osteoporosis with both therapeutic anabolic and anticatabolic benefits, and their potential mechanisms of action.

## Natural Chinese Medicines

### *Gynochthodes officinalis* (F.C.How) Razafim. & B.Bremer (syn. *Morinda officinalis* F.C.How)

*Gynochthodes officinalis* (F.C.How) Razafim. & B.Bremer (syn. *Morinda officinalis* F.C.How) (MO, “Ba-Ji-Tian”) is a famous natural herb in Chinese medicine, containing many bioactive components including oligosaccharides, iridoid glycosides, and bajijiasu (Chen et al., 2014; Wu et al., 2015b; Li et al., 2016a). Recent studies have found that it could enhance sexual and reproductive function; and may ameliorate Alzheimer’s disease *via* the microbiota-gut-brain axis, providing benefits including improved memory and learning abilities (Wu et al., 2015b; Chen et al., 2017a). Additionally, the components of polysaccharides, monotropein and bajijiasu could act as potential agents to treat osteoporosis.

The protective effects of MO root extract on ovariectomy-induced bone loss have been reported (Li et al., 2009). Findings indicate that MO root extract could enhance the bone mineral content (BMC) and bone mineral density (BMD) of the tibia in ovariectomized (OVX) rats (Li et al., 2009). The levels of serum phosphorus (P), calcium(Ca), and osteoprotegerin (OPG) increased, and the levels of serum deoxypyridinoline crosslinks to creatinine ratio (DPD/Cr), tartrate-resistant acid phosphatase (TRAcP), adrenocorticotropin (ACTH), and corticosterone decreased, but did not reverse the levels of serum alkaline phosphatase (ALP), tumor necrosis factor-α (TNF-α), or interleukin-6 (IL-6) (Li et al., 2009). Correspondingly, MO capsules attenuated the ovariectomy-induced loss of bone mass by enhancing bone strength, and reducing further deterioration of the trabecular microarchitecture of the femurs in rats, which was associated with increased serum osteocalcin (OC) levels (Li et al., 2014b). Polysaccharides are the extract isolated from MO that could significantly elevate whole femoral BMD as compared with the osteoporosis group. The mineral levels of Ca, P, Mg, Zn, Mn, Cu, and Fe in the femur were enhanced dose-dependently (Zhu et al., 2008). Monotropein is a natural compound extracted from MO that appears to exhibit antiosteoporotic effects by increasing BMC, BMD, bone volume fraction (BVF), and attenuating trabecular microstructural degradation in OVX rodents (Zhu et al., 2008; Zhang et al., 2016c). In vivo findings from a disuse-model of osteoporosis indicate that MO dried-root extracts exhibit both bone formation activity and appear to suppress bone resorption. Mornidae Radix (MR), the dried-root of MO, was found to inhibit the osteoporosis-induced decrease of hind limb thickness, tibia failure load, BMD, and tibia Ca and P levels (Seo et al., 2005). Histomorphometry results indicated that it could remarkably protect tibiae’s bone parameters, including volume, length, and cortical and trabecular thickness. Furthermore, both preventive and therapeutic studies indicated that the effects of MR appear to be favourable for both the prevention and treatment of osteoporosis (Seo et al., 2005).

Consistently, *in vitro* studies have found that MO and its bioactive components appear to provide potentially therapeutic treatment for osteoporosis, and that the molecular mechanisms appear to act *via* receptor activator of nuclear factor kappa-B ligand (NF-κB, or RANKL) signalling pathways. For example, rubiadin-1-methyl ether (RBM, a natural anthraquinone compound isolated from the root of MO) may effectively inhibit osteoclastic bone resorption by blocking the NF-κB pathway (He et al., 2018). Bajijiasu was found to inhibit osteoclast formation and bone resorption *via* the mediation of RANKL signalling pathways (Hong et al., 2017a). Expression of RANKL-induced marker genes, including cathepsin *K (CTSK)*, nuclear factor of activated T cells cytoplasmic 1 (NFATc1), TRAcP, vacuolar-type H+-ATPase V0 subunit D2 (V-ATPase d2), and matrix metalloproteinase-2 (MMP-2) was inhibited by Bajijiasu (Hong et al., 2017a). Further *in vitro* studies of anthraquinones isolated from MO have identified these molecules as potential inhibitors of osteoclastic bone resorption and enhancers of osteoblastic bone-forming activity (Wu et al., 2009; Bao et al., 2011). MO-anthraquinones have demonstrated the ability to decrease the formation of bone resorption pits, the number of multinucleated osteoclasts, and the activity of tartrate resistant acid phosphates (TRAP) in an osteoblast-bone marrow coculture system, in addition to expediting the apoptosis of osteoclasts (Bao et al., 2011). MO-anthraquinones also appear to promote osteoblastic bone-forming activity by increasing osteoblast proliferation and ALP activity (Wu et al., 2009). Investigation of the polysaccharides extracted from MO revealed that osteogenic inulins of MO promote the proliferation, differentiation, and mineralization of osteoblast cells, as confirmed by the up-regulation of marker genes, including runt-related transcription factor 2, osterix, osteocalcin, bone sialoprotein, and OPG (Jiang et al., 2018).

Taken together, these findings suggest that MO and its bioactive components might provide an important therapeutic role for the treatment and prevention of osteoporosis. MO appears to provide potentially effective treatment for osteoporosis by the attenuation of bone loss and trabecular microstructural degradation, the enhancement of bone mineral density and bone mass, the promotion of osteoblastic bone-forming activity, and the inhibition of osteoclastic bone resorption by direct effects on osteoclasts and effects mediated *via* known signalling pathways. In the *in vitro* research, the effective concentration of Bajijiasu compound was found to be 0.8 mM (Hong et al., 2017a), which may be higher than compounds in other *in vitro* experiments, and the clinical usage may be increased to get better curative effects, therefore, a careful caution of the safety should be observed. Further research is needed to develop the therapeutic potential of this natural medicine.

### *Curculigo orchioides* Gaertn

There are more than 20 species of *Curculigo* plants in the world. They are native to the tropical and subtropical regions of Asia, Africa, South America, and Oceania (Nie et al., 2013). *Curculigo orchioides* Gaertn (CO, “Xian Mao”), one of curculigo species, is used to relieve the symptoms of limb weakness, lumbar and knee joint arthritis, and diarrhea in Chinese medicine (Tan et al., 2019). Recent studies have found that CO also appears to have antioxidant properties and anticancer potential, and may be used as an antiosteoporotic herb (Cao et al., 2008; Ramchandani et al., 2014; Hejazi et al., 2018).

CO is used in Chinese medicine for the treatment of postmenopausal osteoporosis and the antiosteoporotic effects of CO have been investigated *in vivo* (Cao et al., 2008). Administration of CO prevented trabecular bone loss in the tibia of ovariectomized rats by inhibiting bone resorption and increasing serum phosphorus, calcium, and OPG levels, without affecting the body or uterus mass (Cao et al., 2008). The serum levels of bone resorption related makers, DPD/Cr, TRAcP, ACTH, and corticosterone were decreased following CO administration (Cao et al., 2008). CO may also enhance bone formation upon induction during wound healing (Wong et al., 2007a).

Curculigoside (CCG, a phenolic glycoside) is the main bioactive compound of CO and appears to have both osteogenic and antiosteoclastic effects (Liu et al., 2014a). A recent *in vitro* study evaluated the effect of CCG on osteogenic differentiation of human amniotic fluid-derived stem cells (hAFSCs) and found that CCG up-regulated osteogenic activity in a dose-dependent manner, including increased expression of osteopontin (OPN) and Collagen I, increased ALP activity and calcium deposition (Liu et al., 2014a). Furthermore, the ratio of OPG to RANKL increased, indicating that osteoclastogenesis was inhibited. The simultaneous up-regulation of β-catenin and Cyclin-D1 indicate that these effects appear to be mediated *via* the Wnt/β-catenin signalling pathway (Liu et al., 2014a). The *in vitro* effect of CCG on osteogenic differentiation has also been investigated in relation to BMSCs, and the data indicate that CCG may promote the proliferation and osteogenic differentiation of BMSCs (Shen et al., 2013).

The antioxidant properties of CO are also thought to be attributable to CCG, and may play a critical role in attenuating osteoporosis pathophysiology (Wang et al., 2012b). The antioxidant protective effects of CCG have therefore been investigated in rat calvarial osteoblasts exposed to hydrogen peroxide (H_2_O_2_), and findings indicate that CCG significantly reduce the cytotoxic effects of H_2_O_2_ by reducing the production of reactive oxygen species (ROS) by osteoblasts, and recovering the levels of osteoblast differentiation markers, including ALP, calcium deposition, and runt-related protein 2 (Runx2) (Wang et al., 2012b). CCG has also been shown to protect rat calvarial osteoblasts from dexamethasone (DEX) induced cytotoxicity by regulating osteoblast proliferation, differentiation, and proinflammatory cytokine levels (Zhu et al., 2015). The effects of DEX on the levels of osteoblast differentiation markers, including ALP, OPG, β-catenin, and RANKL was reversed, indicating that CCG may be a suitable compound for the treatment of osteoporosis (Zhu et al., 2015). Accordingly, metabolic analysis indicates that CCG *via* its main metabolite, M2, could produce these antiosteoporotic effects (Wang et al., 2017a).

Further *in vitro* and rat calvarial studies have shown that compounds isolated from CO appear to have antiosteoporotic properties, including the promotion of osteoblast proliferation and differentiation, increased bone-forming activity, and the inhibition of osteoclastic bone resorption. The bioactive compounds isolated from MO include novel homogeneous polysaccharides, COP90-1 and COP70-3, and phenolic glycosides (Jiao et al., 2009; Wang et al., 2013b; Wang et al., 2017c; Wang et al., 2019b).

Briefly, the promotion on osteogenesis of C. *orchioides* might be the beneficial effect leading to attenuation of osteoporosis, and the inhibition of reactive oxygen species is a great property of this medicine. Additionally, some compounds isolated from CO exhibit the suppression of osteoclastic bone resorption. Further research is necessary to discover the molecular mechanisms, signalling pathways, and bioactive constituents of CO; and to determine the molecular basis of the potential relationship between the antioxidant effects of CCG and osteoporosis pathophysiology in osteoclasts and osteoblasts.

### *Psoralea corylifolia* (L.) Medik

*Psoralea corylifolia* (L.) Medik (PC, “Bu Gu Zhi”) is widely used in Asia for medicinal purposes; the dried ripe seeds are the active component of the plant. PC is used in Chinese pharmacopoeia and TCM formulas for the treatment of skin diseases, cardiovascular disease, nephritis, and osteoporosis (Zhang et al., 2016a). The primary bioactive compounds of PC are coumarins (e.g., psoralen), flavonoids (e.g., neobavaisoflavone, bavachalcone, bavachin, and corylin), and meroterpenes (e.g., bakuchiol) (Zhang et al., 2016a). Additional components of PC have immunoregulatory, antioxidant, and neuroprotective properties for the treatment of Parkinson’s disease (Jing et al., 2017). PC may also have therapeutic potential for the treatment of prostate cancer (Lin et al., 2018).

The potential beneficial effects of PC in relation to osteoporosis treatment has been evaluated in animal models (Miura et al., 1996; Tsai et al., 2007). Administration of PC was found to decrease urinary calcium secretion and decrease serum calcium in OVX rats, resulting in improvements in BMD and bone formation, and may have therapeutic potential for the treatment of postmenopausal osteoporosis (Tsai et al., 2007). Complementary investigation indicates that PC may have therapeutic benefits for the broader treatment of bone pathological conditions, including bone fracture, osteomalacia, and osteoporosis (Miura et al., 1996). Furthermore, two compounds of PC, bavachin (BA, a flavonoid), and bakuchiol (BK, a meroterpene) have been shown to have osteoanabolic activity by inducing osteoblast differentiation. And maybe bioactive components of PC that could provide protection against post-menopausal bone loss (Lim et al., 2009; Weng et al., 2015). Bavachin appears to be more effective in stimulating cell proliferation, whereas bakuchiol appears to have a greater effect on osteoblast differentiation (Li et al., 2014a). The molecular structures of bavachin and bakuchiol that hypothetically produced variation in the osteogenic effects between these two and additional compounds of PC depending on its position, is a prenyl group side chain (Li et al., 2014a). Further research is needed to investigate the effect of changing the position of the prenyl group on the strength of osteoblast activity of PC compounds (Li et al., 2014a). The osteogenic effects of BA and BK appear to be produced by up-regulation of the Wnt signalling pathway, and produce increased ALP levels, Ca serum concentration, and BMD (Lim et al., 2009; Weng et al., 2015). Further *in vitro* findings suggest that BK inhibits RANKL-induced osteoclast differentiation and bone resorption by disrupting the AKT and AP-1 signalling pathways (Chai et al., 2018).

Psoralen (PSO), an active coumarin-compound of PC, is reportedly a promoter of bone mass and has been tested in a rodent ovariectomized model of osteoporosis (Yang et al., 2012). Findings showed that PSO produced an increase in trabecular thickness, and up-regulated osteogenic markers, including osteocalcin and ALP (Yang et al., 2012). Furthermore, the *OVX*-induced gene expression profile was reversed by PSO treatment, appearing to be mediated *via* the Notch signalling pathway, and resulted in regulation of the differentiation of bone mesenchymal stem cell, thus indicating the osteogenic potential of PSO for the treatment of postmenopausal osteoporosis (Yang et al., 2012). In vitro findings indicate that PSO may also attenuate osteoclast differentiation and bone resorption *via* inhibition of the AKT and AP-1 signalling pathways (Chai et al., 2018).

Neobavaisoflavone (NBIF), an isoflavone compound of PC, may also have an osteogenic activity that could be applied for the treatment of bone fractures, osteomalacia, and osteoporosis (Don et al., 2012). In vitro findings demonstrated that NBIF enhanced osteogenesis in a concentration-dependent manner by increasing ALP activity, increasing the expression of bone-specific matrix proteins (including type I collagen, Col-I), increasing osteocalcin and bone sialoprotein levels, up-regulating the expression of Runx2 and osterix (OSX), and by the formation of bone nodules (Don et al., 2012). Further experiments indicate that inhibition of the p38 receptor results in the inhibition of NBIF osteogenic activity, and, that NBIF also increases the active phosphorylated level of p38 in a concentration-dependent manner. Therefore, the osteogenic activity of NBIF appears to be mediated by activation of the p38-dependent signalling pathway (Don et al., 2012).

The flavonoid compound, bavachalcone, isolated from PC may be an effective herbal compound to inhibit osteoclast activity and attenuate osteoporosis-induced bone loss (Park et al., 2008). Bavachalcone was found to inhibit osteoclast formation of primary culture osteoclast precursor cells, *in vitro* (Park et al., 2008). In the presence of bavachalcone, the activity of the osteoclast differentiation factor, NFκB (RANKL), was reduced, leading to inhibition of osteoclastogenesis *via* suppression of transcription factors, c-Fos, NFATc1, and by blocking MEK, ERK, and Akt signalling (Park et al., 2008).

Taken together, these findings suggest that *P. corylifolia* appears to be an important therapeutic agent in Chinese medicine for the treatment of osteoporosis. The compound of bakuchiol should be studied more deeply for it appears to have dual properties of promoting osteogenic activity and ameliorating osteoclastic bone resorption to attenuate osteoporosis, and it has a higher promotion on osteoblast differentiation than bavachin (Li et al., 2014a; Chai et al., 2018). Further research is needed to isolate, characterize, and investigate the effect of variation of the molecular structure of the active compounds of PC on osteoblast and osteoclast activity, including their biochemical effects and signalling pathways.

### *Eucommia ulmoides* Oliv.

*Eucommia ulmoides* Oliv. (EU, “Du Zhong”) is a plant, and the dried bark of EU is used in TCM and as a food source (Hussain et al., 2016). EU has pharmacological properties as an antioxidant, anti-inflammatory, antimicrobial, anticancer, cardioprotective, and neuroprotective agent that have been applied for the treatment of cardiovascular disease, sexual dysfunction, cancer, metabolic disease, neurological disease, rheumatoid arthritis, osteoarthritis, and diabetes (Xie et al., 2015; Hussain et al., 2016; Wang et al., 2016a; Do et al., 2018). The bioactive compounds of EU include lignans, iridoids, phenolics, steroids, and flavonoids (Hussain et al., 2016). Additional studies have found that the EU could also be effective in the treatment of osteoporosis.

Du Zhong cortex extract (DZCE) has been evaluated to investigate the potential protective benefits against lead-induced, estrogen deficiency-induced, and disuse-induced osteoporosis (Zhang et al., 2009; Pan et al., 2014; Qi et al., 2019). In a rodent model of lead acetate-induced bone loss, DZCE attenuated the loss of BMD of the lumbar spine and femur, and restored serum calcium, phosphorous, ALP, osteocalcin, and RANKL to normal levels (Qi et al., 2019). Furthermore, antiosteoclastic activity was indicated by the effect of DZCE on adjusting the serum OPG/RANKL ratio to normal values (Qi et al., 2019). In an OVX-rat model of estrogen deficiency-induced osteoporosis, higher doses of DZCE prevented further deterioration of the biomechanical properties of the femur, including maximum stress and Young’s modulus, which was accompanied by attenuation of loss of BMD (Zhang et al., 2009). These findings were supported by micro-CT analysis indicating that the parameters of BMD and bone thickness were improved by higher doses of DZCE, and because the levels of bone turnover markers, osteocalcin, ALP, and deoxypyridinoline (DPD) were decreased (Zhang et al., 2009). Total lignans (TL) extracted from EU-cortex, was also found to inhibit the loss of bone mass due to estrogen deficiency-induced osteoporosis in a rodent model (Zhang et al., 2014). In vivo findings demonstrated improvements in the biomechanical quality of the femur, in terms of maximum stress and Young’s modulus, and micro-CT analysis showed prevention of further trabecular microstructural degradation (Zhang et al., 2014). In vitro findings showed that TL promoted the proliferation and differentiation of osteoblasts; and inhibited osteoclastogenesis, by an increase in OPG and decrease in RANKL expression (Zhang et al., 2014). In a rodent disuse-induced osteoporosis model, DZCE treatment improved bone strength and prevented trabecular microstructural degradation, and reduced the levels of bone turnover markers, including TRAcP, DPD, and OC (Pan et al., 2014). Additional findings in rodents indicate that EU could promote longitudinal bone growth by increasing chondrogenesis of the tibial growth plate, and increasing levels of BMP-2 and IGF-1 (Kim et al., 2015). Taken together, these results suggest that DZCE may provide effective treatment for osteoporosis by attenuating bone loss, both by the formation of bone, and by inhibiting osteoclastic bone resorption; and may promote longitudinal growth of the long bones.

5-(hydroxymethyl)-2-furaldehyde (5-HMF) is a bioactive compound isolated from Eucommiae Cortex that has been shown to promote osteogenesis and inhibit adipogenesis (Tan et al., 2014). In vitro findings demonstrated that 5-HMF induction of BMSCs in normal medium up-regulated the expression of osteogenesis-markers (ALP, COL1alpha1, OC, and OPN), and that 5-HMF decreased the expression of adipogenesis-markers (PPARγ, FABP4, C/EBPα, and LPL) by BMSCs in adipogenic induction medium (Tan et al., 2014). Furthermore, mineralized nodule formations were produced by BMSCs induced by 5-HMF cultured in both normal and adipogenic-inducing medium, indicating the potent osteogenic, antiadipogenic, and antiosteoporotic properties of 5-HMF (Tan et al., 2014). Further research is necessary to investigate the molecular mechanisms involved with the osteogenic, and potential pro-osteoblastic and antiosteoclastic effects of 5-HMF and additional bioactive components of EU-cortex (Ha et al., 2003).

The leaves and seeds of EU may also be of potential benefits for the treatment of osteoporosis (Li et al., 2011; Zhang et al., 2012). In a combined estrogen deficiency-induced osteoporosis and obesity rodent model, Eucommia leaf extract (ELE) treatment decreased body weight and BMI, and increased tibial and femoral BMD, and increased bone strength, which appeared to be the effects of restoring bone metabolism, respective of bone formation, and adsorption (Zhang et al., 2012). Further rodent study indicates that administration of total glycosides from Eucommia ulmoides seed (TGEUS) could enhance the BMD and the microarchitecture parameters of the femur in healthy rats (Li et al., 2011). Iridoid compounds isolated from the leaves of the EU may be the bioactive components that render the therapeutic effects (Takamura et al., 2007).

Taken together, we think that the herb of *E. ulmoides* is a great medicine to treat bone loss diseases, for many parts of EU including the cortex, leaf, and seed, might have potential therapeutic benefits for the treatment of osteoporosis, as depicted above (Li et al., 2011; Zhang et al., 2012; Qi et al., 2019). Furthermore, *in vivo* and *in vitro* findings show that they provide effective treatment for osteoporosis by increasing bone strength and preventing trabecular microstructural degradation, promoting osteogenesis, and inhibiting bone resorption. Usually, the cortex is the major part of the EU that is used to treat bone, cardiovascular, and sexual diseases. Based on the positive evidence and effects of the leaf and seed, more research is needed to isolate and characterize the bioactive molecules of EU, and compare the effects between cortex, leaf, and seed for the treatment of osteoporosis, including the mechanisms that produce the potential therapeutic effects among them.

### *Dipsacus inermis* Wall.

*Radix Dipsaci* (RD, “Xu Duan”) is the dried roots of *Dipsacus inermis* Wall., and it is used in Chinese medicine to strengthen bone (“Xu Duan” means “to promote the growth of bones to correct bone fractures”). Recently, studies have reported that RD may be a beneficial bone formation agent to treat osteoporosis (Liu et al., 2009; Niu et al., 2012; Liu et al., 2012b).

The bone formation properties that may provide therapeutic benefit for the treatment of osteoporosis have been investigated in recent animal studies. In ovariectomy-induced postmenopausal rodent models, RD treatment prevented the loss of bone mass and trabecular microstructural degradation, and improved bone strength (Liu et al., 2009; Liu et al., 2012b). These results may be attributed to altered bone remodeling, as evidenced by a decrease in the level of bone turnover markers, including ALP, OC, and DPD. Furthermore, the effects of ovariectomy on OPG and RANKL levels of osteoblasts and BMSCs were reversed by treatment with RD (OPG levels increased and RANKL levels decreased), indicating the osteogenic effect of RD on molecular biomarkers (Liu et al., 2012b). The osteogenic effect of the dichloromethane fraction of RD on BMSCs has been demonstrated *in vitro* by the formation of calcified nodules, increased ALP activity, increased expression of bone sialoprotein (BSP) and OC (Kim et al., 2011). An active single compound isolated from the dichloromethane fraction of RD, hederagenin 3-O-(2-O-acetyl)-α-L-arabinopyranoside, significantly increased ALP, OC, and BSP levels (Kim et al., 2011). A hindlimb unloading rodent model of osteoporosis investigated the effect of RD treatment on BMD and bone microarchitecture, and showed that the biomechanical properties were enhanced (Niu et al., 2015a). Oral administration of RD improved the mechanical strength of bone, BMD, BMC, and bone turnover markers, including urinary calcium and phosphorus excretion. Micro-CT analysis showed that RD prevented trabecular microstructural degradation, improved the bone volume fraction, and improved tissue mineral density and content (Niu et al., 2015a). Healthy rats fed with Radix Dipsaci extract (RDE) showed an anabolic systemic skeletal effect by increased bone density and altered bone histomorphology (Wong et al., 2007b). An increase of bone trabeculae of 11.82% was measured, indicating that bone density was increased, and the bone histomorphology has also been improved by the enhancement of BV/TV (increased 4.5%), and improvements in BS/TV, Tb.N, and the reduction in Tb.Sp (Wong et al., 2007b). Taken together, these findings suggest that RD may provide beneficial therapeutic effects for the treatment of osteoporosis.

Further studies of the bioactive components of RD indicate that several compounds, including saponins and iridoid glycosides extracted from RD and may produce biological effects (Kim et al., 2011; Tao et al., 2019). The antiosteoporotic effect of RD total saponins (RTS) may be related to its effect on osteoblast and osteoclast cells (Niu et al., 2012; Niu et al., 2015b). Oral administration of RTS has been shown to prevent OVX-induced loss of bone mass in rats, indicated by decreased levels of bone turnover markers, including urinary calcium, and phosphorous excretion, and by increasing the biomechanical strength of bone and preventing trabecular microstructural degradation (Niu et al., 2012). In vitro findings determined that RTS promoted osteoblastic cell maturation and differentiation, increased ALP and OC levels, and increased the synthesis of BMP-2, leading to increased bone formation (Niu et al., 2012; Niu et al., 2015b). The positive effect of RTS on osteoblast cells appears to be mediated *via* the BMP2/mitogen-activated protein kinase (MAPK)/Smad1/5/8-dependent Runx2 signalling pathway (Niu et al., 2015b). Furthermore, RTS inhibited osteoclastogenesis by increasing the expression of OPG and decreasing the expression of NF-κB (RANKL) (Niu et al., 2012). Additionally, asperosaponin VI is a saponin isolated from RD that has been shown to promote the proliferation, differentiation and mineralization of osteoblastic cells, and these effects appear to be mediated by BMP-2 synthesis, and activation of p38 and ERK1/2 signalling (Niu et al., 2011).

Collectively, these findings suggest that RD appears to provide beneficial therapeutic effects for the treatment of osteoporosis, including attenuating bone loss and increasing bone strength and quality, that may be attributed to their improved physiological bone remodelling *in vivo*. At cellular and molecular levels, the RD and its bioactive components appear to promote osteoblast differentiation, proliferation, maturation, and mineralization, and may inhibit osteoclastogenesis. The bone research-related amimal models were rich in these studies including ovariectomy-induced postmenopausal rodent models, hindlimb unloading model, and healthy rats to detect the bone metabolism. While some treatment ingredients of Radix Dipsaci, such as ethyl alcohol or crude extractions, are not well studied. It is necessary for further research to identify the efficient compounds and to develop the therapeutic potential and mechanism of RD in the treatment of osteoporosis.

### *Cibotium barometz* (L.) J.Sm

*Cibotium barometz* (L.) J.Sm (CB, “Gou Ji”) is well-known in TCM and its hairs are a staple ingredient used in ointments, such as an antihaemorrhagic agent for wound healing poultices (Wu and Yang, 2009). The rhizome of CB contains anti-inflammatory properties and is used in the treatment of diseases including rheumatism, lumbago, sciatica, and dysuria in the aged (Wu and Yang, 2009). CB is thought to have properties that nourish bone and improve gonadal function. And it is frequently used in herbal remedies for the treatment of osteoporosis (Zhao et al., 2011).

Due to the potential benefits of CB for the treatment of osteoporosis, research has been performed to determine the effects of CB *in vivo* and *in vitro*. In a rodent model of post-menopausal estrogen-deficiency-induced osteoporosis, daily administration of the extract of CB (CBE) was shown to prevent femur total BMD loss in OVX-rats, which appeared to be associated with a decrease in skeletal remodeling, as evidenced by decreased levels of bone turnover markers OC, ALP, DPD, and urinary excretions of calcium and phosphorous (Zhao et al., 2011). Furthermore, the CBE treatment also appeared to enhance bone strength and prevent trabecular microarchitectural degradation as determined by improvements in micro-CT microstructural parameters. When compared with the untreated model rats, CBE treatment significantly increased the bone maximum stress, energy, and Young’s modulus of OVX rats (Zhao et al., 2011). CBE treatment performed favourably in comparison with estrogen-therapy in terms of its effect on body weight and uterine weight (Zhao et al., 2011). Additional *in vitro* experiments have been performed to evaluate the potential effectiveness of the constituents of the rhizome of CB in the treatment of osteoporosis (Nguyen et al., 2009; Xu et al., 2014). Findings from these experiments indicate that these CB-rhizome constituents could promote the proliferation and differentiation of rat osteoblasts, and thus be potential therapeutic agents for the treatment of osteoporosis (Xu et al., 2014). Furthermore, compounds isolated from CB-rhizome appeared to inhibit osteoclastogenesis from primary bone marrow macrophages (BMMs) when cultured in an inductive medium, without any adverse effects on the viability of precursor BMM cells (Nguyen et al., 2009).

Therefore, evidence suggests that CB could be used as an effective therapeutic agent for the treatment of osteoporosis. The ingredients of CB in some research were still not very clear or definite, which are obtained by ethanol extraction from the dried and crude *C. barometz* (Zhao et al., 2011). Further research is needed to isolate and characterize the bioactive constituents of CB, and to determine the molecular mechanisms and signalling pathways by which they produce therapeutic effects.

### Velvet Antler

*Velvet Antler* (VA, “Lu Rong”) is the precalcified cartilaginous antler, in TCM commonly obtained from the Silka deer and Red deer, that is used as an effective agent to strengthen bone and for the improvement of immune health, physical strength, and sexual function (Zhang et al., 2013; Sui et al., 2014). Velvet antlers regenerate annually in mammals, and ingestion of VA is thought to confer similar benefits upon the consumer (Gilbey and Perezgonzalez, 2012). Both VA and its close relative, deer antler base, are designated medicinal products in the Chinese Pharmacopoeia (Wu et al., 2013). The bone-health promoting properties of VA has been investigated in relation to the treatment of bone diseases including osteoporosis and osteonecrosis (Zhang et al., 2013; Wei et al., 2016).

The effectiveness of VA for the treatment of postmenopausal estrogen deficiency-induced osteoporosis has been investigated using rodent models (Yang et al., 2010a; Tseng et al., 2012; Zhang et al., 2013). Total velvet antler polypeptides (TVAP) administered to OVX-rats were found to prevent bone loss and improve BMD, BMC, and bone microarchitecture, which appeared to be associated with inhibition of IL-1 and Il-6 (Zhang et al., 2013). Furthermore, natural velvet antler polypeptides (nVAP) and synthetic-VAP (sVAP) promoted the proliferation of cartilage and osteoblast-like cells, and inhibited the activity of IL-1α secreted from THP-1 monocytic cells *in vitro*, indicating that TVAPL and sVAP may be potential therapeutic agents for the treatment of postmenopausal osteoporosis (Zhang et al., 2013). Both VA and VA-blood can be used in TCM to tonify and invigorate bones and tendons, and the combination of middle sections of VA and VA-blood (VAM-B) is thought to have superior pharmacological effects (Tseng et al., 2012). In OVX-rats, treatment with VAM-B improved body weight, and increased the strength of the vertebra and femur, and improved tibial trabecular microarchitecture, which was accompanied by a decrease in ALP levels. The results supported the therapeutic use of VAM-B for the treatment of postmenopausal osteoporosis (Tseng et al., 2012). Additional research has shown that oral administration of VA-blood to OVX-rats could attenuate reduced BMD of the lumbar vertebra and femur, and normalize serum levels of insulin-like growth factor -1 (IGF-1) and testosterone (Yang et al., 2010a).

Interestingly, the effect of fermentation on the bone formation capability of VA was investigated *in vitro* (Lee et al., 2011). Findings indicated that both non-fermented antler (NFA) and fermented antler (FA) treatment increased the proliferation of osteoblasts and bone matrix proteins, including type I collagen and BSP (Lee et al., 2011). Moreover, FA showed enhanced osteoblast proliferation, increased ALP activity, and increased mineralization compared to NFA and, therefore, the fermentation process may enhance the bone-forming effects of antler (Lee et al., 2011). Furthermore, the effect of fermentation on antiresorptive activity was investigated *in vitro*, and it appears that the fermentation process may improve the capability of VA to inhibit osteoclast differentiation and signalling activity (Choi et al., 2013). Findings indicate that the extract of FA inhibited RANKL-induced osteoclast differentiation from BMMs by downregulating the expression and activity of NFATc1, which was associated with inhibition of phospholipase Cγ2 (PLCγ2), a signalling molecule known to affect NFATc1 transcriptional activity (Choi et al., 2013). Thus, research suggests that FA extract may inhibit osteoclastic activity *via* disrupting PLCγ2-NFATc1 signalling, and may provide therapeutic benefit for the treatment of osteoclast-related bone diseases, including osteoporosis (Choi et al., 2013). Furthermore, *in vitro* studies have shown that the chloroform extract (CE-C) of deer antler appears to inhibit osteoclast differentiation by suppressing the activation of extracellular signal-regulated kinase (ERK), protein kinase B (PKB/Akt), the inhibitor of kappa B (I-κB), which would be increased by RANKL under osteoporosis conditions (Li et al., 2007). CE-C also appears to inhibit the bone resorption activity of osteoclasts and disrupt the actin rings, leading to osteoclast apoptosis (Li et al., 2007). Taken together, these findings suggest that VA and CE-C may be effective compounds for the treatment of osteoporosis.

Further research has compared the osteogenic capacity of VA from different sections of antler on longitudinal bone growth and osteoporosis (Tseng et al., 2014; Kim et al., 2016). In vivo and *in vitro* findings indicate that VA promotes longitudinal bone growth in adolescent rats through enhanced BMP-2 expression, osteogenic gene expression, including collagen, ALP, and OC, and by promoting the proliferation, differentiation, and mineralization of osteoblast-cells (Kim et al., 2016). The osteogenic effects of VA appear to decrease from the upper or distal sections to the basal sections of antler (Tseng et al., 2014; Kim et al., 2016).

Taken together, these findings indicate that VA appears to contain properties to strengthen bone, promote bone growth, and may provide therapeutic benefit for the treatment of osteoporosis by the bone formation and antiresorptive activity. Interestingly, research by Kim and Tseng et al. indicates that the basal sections of antler should be the prior selection for the better osteogenic effects when applied for the treatment of osteprorosis clinically. Further research is needed to identify and characterize the bioactive components of different sections of VA and the molecular signalling mechanisms that mediate their therapeutic effects, under the influence of varying preparation conditions.

### *Cistanche deserticola* Y.C.Maf

*Cistanche deserticola* Y.C.Maf (CD, “Rou Cong Rong”) is used in TCM for the treatment of kidney deficiency, sexual dysfunction, female infertility, and constipation (Fu et al., 2018). Recent reports suggest that CD could promote male fertility, and may attenuate tinnitus in patients with chronic nephritis, in addition to providing effective treatment for osteoporosis (Jiang et al., 2016; Fu et al., 2018; Fan et al., 2019).

*In vivo* studies using rodent models of postmenopausal estrogen deficiency-induced osteoporosis have shown that Cistanches deserticola-extract (CDE) could provide therapeutic benefits for the treatment of osteoporosis (Liang et al., 2011; Liang et al., 2013). Findings indicate that CDE could dose-dependently enhance the femoral BMD and BMC in OVX rats, and improve biomechanical femur parameters of loading and autobreak including maximum load, maximum displacement, and stress (Liang et al., 2011). In comparison with OVX rats, CDE-treatment rats showed biochemical differences including decreased blood calcium, zinc, and copper levels (Liang et al., 2011). Further *in vivo* investigation of the molecular mechanisms behind the antiosteoporosis effect of CDE indicate that the attenuation of bone degeneration is associated with the regulation of genes involved with bone metabolism, including *Smad1*, *Smad5*, *TGF-β1*, and *TIEG1* (Liang et al., 2013). Additionally, *in vivo* investigation of the effects of CD compound, Cistanoside, on OVX rats indicates that CD may contain both osteogenic and antiosteoclastic properties (Xu et al., 2017). In addition to increasing bone strength, BMD, and improving trabecular microstructure, Cistanoside may also decrease the activity of bone resorption markers including TRAP, DPD, and cathepsin K (Xu et al., 2017). These effects appear to be mediated by down-regulation of TNF-receptor associated factor 6 (TRAF6), which downstream mediates both the inactivation of NF-κB, to inhibit osteoclast activity, and the stimulation of the PI3K/Akt osteogenic pathway (Xu et al., 2017).

*In vitro* studies have demonstrated that CD may induce bone formation for the treatment of osteoporosis (Li et al., 2012a; Li et al., 2012b). CD extract increased the expression of ALP, BMP-2, and OPN, and increased bone mineralization by cultured osteoblast cells (Li et al., 2012b). Echinacoside (ECH), a phenolic compound isolated from CD, appears to promote bone formation activity by cultured osteoblast cells, including increased cell proliferation, ALP activity, OC levels, and mineralization, which may be associated with increasing the OPG/RANKL ratio (Li et al., 2012a). Correspondingly, *in vivo* findings support the positive dose-dependent effects of ECH for the treatment of osteoporosis in OVX rats (Li et al., 2013a). Following ECH treatment for 12 weeks at the doses of 90 mg/kg and 270 mg/kg, the femur BMD was significantly enhanced (Li et al., 2013a). Furthermore, the histomorphological and micro-CT analysis indicated that the deteriorated microarchitecture and biomechanical parameters had been reversed, and that the cortical bone thickness, osteoblasts number, and trabecular thickness were enhanced (Li et al., 2013a). Further research suggests that CD derived compounds may also decrease osteoclastic bone resorption by inhibiting osteoclastogenesis, attenuating RANKL activity, and disabling NFAT and MAPK (Song et al., 2018).

Collectively, these findings suggest that CD may contain properties that are potentially effective for the treatment of postmenopausal osteoporosis. Though the compound of Cistanoside may act as a good representative of *Cistanches deserticola* with the interesting study that it could possess both osteogenic and antiosteoclastogenesis properties, further research is necessary to isolate and characterize the bioactive constituents of CD and to investigate their potential osteogenic and antiresorptive effects, and the molecular signalling pathways affected by Cistanches herb in relation to osteoporosis.

### *Cuscuta chinensis* Lam.

The seed of *C. chinensis* Lam. (CC, “Tu Si Zi”) is one of the commonly used herbs in Chinese medicine for the enhancement of sexual function, vision, and birth control (Donnapee et al., 2014). The biological activity of CC includes skin and liver protection, immune regulation, neuroprotection, antioxidant, and anti-inflammatory properties (Donnapee et al., 2014). Current research is investigating the effectiveness of CC for the treatment of osteoporosis (Donnapee et al., 2014).

*In vivo* and *in vitro* experiments indicate that the bioactive compounds of CC that appear to be effective against osteoporosis are kaempferol, hyperoside, and campesterol (Yao et al., 2005; Yang et al., 2011; Nowak et al., 2017). In a rodent model of postmenopausal estrogen deficiency-induced osteoporosis, kaempferol (isolated from the seeds of CC), increased the femoral BMD and Young’s modulus of OVX rats as compared to untreated controls, which was accompanied by reduced bone turnover, increased bone tissue volume ratio and increased trabecular bone perimeter (Nowak et al., 2017).

*In vitro* studies showed that the extract of CC could dose-dependently increase osteoblast-like cell proliferation and mineralization, and that this was accompanied by enhancing ALP activity, increased collagen synthesis, and BMP-2 expression (Yang et al., 2009). Furthermore, CC appears to protect osteoblast cells from tertiary butyl hydroperoxide (TBHP) oxidative stress-induced apoptosis, possibly due to its antioxidant activity and function *via* mitochondria-dependent pathways (Gao et al., 2013). Additional *in vitro* evidence has demonstrated that CC may promote the proliferation and differentiation of osteoblast from their precursor cells to induce mineralized nodule formation and decrease osteoclastic activity as indicated by TRAcP (Yao et al., 2005).

Taken together, these findings indicate that the osteogenic, antioxidant, and antiosteoclastic properties of CC may provide therapeutic benefit for the treatment of osteoporosis. Although the evidence has indicated the promotion on osteogenesis and the inhibition on bone resorption by the compounds, kaempferol, and campesterol, their respective functional mechanisms were still not well studied, not like the researches in Yang et al. (2009) and Gao et al. (2013). However, the compounds in Yang and Gao *et al.* were the aqueous extraction, the certain compound were not well known, therefore, further research is necessary to isolate and characterize the bioactive components of *C. chinensis* including their therapeutic potential and molecular mechanisms for the treatment of bone diseases, including osteoporosis.

### *Cnidium monnieri* (L.) Cusson

*Cnidium monnieri* (L.) Cusson (CM, “She Chuang Zi”) is a commonly used herb in Chinese medicine to alleviate pain and inﬂammation, improve sexual potency, for the treatment of skin-related diseases and to improve bone strength (Li et al., 2015c). Over 300 compounds have been isolated from CM and the main bioactive constituents appear to be coumarins compounds (Li et al., 2015c). Further research has reported the potential application of CM for the treatment of Parkinson’s disease, cancer, and osteoporosis (Zhang et al., 2007b; Hong et al., 2017b; Wang et al., 2019c).

Coumarins extracted from CM have been shown to produce potentially beneficial therapeutic effects for the treatment of osteoporosis (Meng et al., 2004; Zhang et al., 2007b; Ming et al., 2013). The chloroform fraction of CM was found to promote osteoblast activity and three coumarins (osthole, bergapten, and imperatorin) were subsequently isolated for further analysis (Meng et al., 2004). In neonatal rodent calvaria culture, osthole was shown to promote osteoblast proliferation and ALP activity, and to inhibit bone resorption by decreasing the formation, differentiation, and TRAcP activity of rat marrow osteoclasts, thus indicating that CM may have potential therapeutic benefits for the treatment of osteoporosis (Meng et al., 2004).

Further *in vivo* and *in vitro* investigations of the potential effectiveness of osthole for the treatment of osteoporosis have been performed (Zhang et al., 2017b). Analysis of findings from a mouse femur fracture model indicated that osthole enhanced fracture repair, bone regeneration, and increased bone strength as compared to untreated controls (Zhang et al., 2017b). In vitro findings determined that osthole promoted osteogenesis by osteoblast cells in a time- and concentration-dependent relationship by increasing osteogenic differentiation, ALP activity, and calcium nodule formation (Ming et al., 2013; Zhang et al., 2017b). In addition, osthole appeared to induce osteogenesis *via* the BMP-dependent signalling pathway as determined by the increased expression of osteogenic-related genes *BMP-2*, *Runx2*, *osterix (Osx)*, and *OCN*, and by the observed inhibition of osteogenesis resulting from antagonization by the BMP-antagonist, noggin (Ming et al., 2013; Zhang et al., 2017b). Further mechanistic analysis demonstrated that the osteogenic effects of osthole appear to be mediated *via* activation of the cAMP/CREB signalling pathway, which appeared to target the transcription factor, Osx (Zhang et al., 2017b). Additional *in vivo* evidence suggests that osthole may provide therapeutic benefit for the treatment of postmenopausal osteoporosis, that may be as effective as 17β-estradiol (Li et al., 2002). In OVX rats, both osthole and 17β-estradiol inhibited estrogen deficiency-induced cancellous bone loss, and increased the biomechanical maximal load of the femoral neck (Li et al., 2002). Osthole could also suppress the urinary deoxypyridinoline (DPD) level of OVX rats. However, osthole did not appear to reduce serum OC levels, nor effects on body or uterus weight as was observed in the 17β-estradiol group, thus indicating that the therapeutic benefits of osthole for the treatment of postmenopausal osteoporosis may not be mediated by the estrogen pathway (Li et al., 2002). Additionally, in healthy rats, osthole was shown to increase total body and femur BMD, trabecular microstructural parameters, and biomechanical properties of maximum load and Young’s modulus, which were accompanied by increased serum OC and TRAcP levels (Cheng et al., 2014). Taken together, these findings suggest that osthole may provide potential therapeutic benefit for the treatment of osteoporosis by promoting osteogenic activity and attenuating bone resorption. Further research is necessary to characterize the antiosteoporosis effects of osthole more extensively.

*In vitro* evidence indicates that imperatorin may also have anabolic potential by stimulating the proliferation and osteogenic activity of MCF-7 cells, and ALP activity of Saos-2 osteoblast cells, possibly by exerting estrogenic properties *via* the estrogen-receptor pathway (Jia et al., 2016). Further research is necessary to investigate the potential therapeutic effects for the treatment of osteoporosis *in vivo*.

Bergapten has also been tested for the potential biopharmacological properties that may be of therapeutic value for the treatment of bone disease, including osteoporosis (Chen et al., 2019a). In vivo investigation of the potential effects of bergapten against ovariectomy-induced osteoporosis in mice, and *in vitro* analysis of RANKL-induced osteoclastogenesis was performed (Chen et al., 2019a). Findings indicated that bergapten appears to inhibit osteoclastic bone resorption and attenuates RANKL-induced osteoclastogenesis, which is mediated *via* disruption of the NF-κB and JNK signalling pathways (Chen et al., 2019a). Furthermore, bergapten effectively reduced the activity of NFATc1 and c-fos (osteoclastogenesis associated transcription factors) which decreased the expression of osteoclast differentiation-related genes, and attenuated osteoclastogenesis by BMMs and RAW264.7 cells without any cytotoxic side-effects. Moreover, bergapten disrupted the formation of F-actin rings, which are implicated in bone resorption activity (Chen et al., 2019a).

Overall, *in vivo* and *in vitro* evidence suggests that coumarin compounds isolated from CM, mainly osthole and bergapten, may produce bio-pharmacological effects for the treatment of osteoporosis by acting to increase bone formation and to decrease bone resorption. Interestingly, the level of serum OC was not affected in OVX rats, while it was enhanced by osthole in the healthy rats, indicating that more studies and attention should be paid to the effects of osthole on serum OC. Further research is necessary to extensively characterize the bioactive effects of CM that render potential therapeutic benefit for the treatment of osteoporosis.

### *Epimedium brevicornum* Maxim

*Epimedium brevicornum* Maxim (EBM, “Yin Yang Huo”) is a very popular natural drug been traditionally used to treat bone diseases, pregnancy, and gonad dysfunction in Chinese medicine for thousands of years. It could relieve postmenopausal symptoms and inhibit osteoporosis and other bone loss diseases, while few hyperplastic effects on the uterus were found. These antiosteoporotic effects may be related with the estrogenic properties by the intrinsic phytoestrogens including some of the flavonoids, lignans, sterols, etc. (Wang et al., 2007; Xu et al., 2016). In the systems pharmacology study, there are 77 components in *Epimedium* possessing the analogous structure to estrogen (Xu et al., 2016). Many of these phytoestrogenic compounds have the beneficial effects to inhibit osteoporosis, including icariin, epimedin A, epimedin B, epimedin C, icariside II and icaritin, epimedoside C, baohuoside I, baohuoside II, etc. (Meng et al., 2005; Huang et al., 2007; Zhai et al., 2013; Liu et al., 2014b; Wang et al., 2016b; Liu et al., 2017b). Among these ingredients, icariin is the main compound of *Epimedium brevicornum* Maxim. Now, there have been many studies and reviews focusing on its anabolic and anticatabolic effects. Certain studies found that icariin has better antiosteoporotic effects than other compounds (Ma et al., 2011; Wang et al., 2018). This review would emphatically introduce the potential effects of icariin to treat osteoporosis, being represented for *Epimedium brevicornum* Maxim.

Icariin, a prenylated flavonol glycoside, was one of the main effective compounds in *Epimedium*. With the instinct estrogen biosynthetic effect (Yang et al., 2013), it had potential osteogenic and antiosteoclastogenic effects *in vitro* and *in vivo*, and antiosteoporotic effects in clinical.

Recent *in vitro* studies have demonstrated that icariin could enhance the ALP activity, osteogenic differentiation and improve the maturation and mineralization of MSCs and osteoblasts including hFOB 1.19 cells, MC3T3-E1, UMR 106 cells (Chen et al., 2007b; Mok et al., 2010; Fan et al., 2011; Cao et al., 2012; Liang et al., 2012). Icariin could also have a pronounced ability to promote the differentiation of osteoblast even with the absence of dexamethasone (Ma et al., 2013). Correspondingly, the mRNA expression of osteogenesis-related genes including *COL1a2*, *OSX*, *RUNX-2*, *BMP-2*, *Smad4*, *Notch2*, and *OPG/RANKL* ratio were significantly increased (Xiao et al., 2005; Zhao et al., 2008; Hsieh et al., 2010; Ma et al., 2011; Bian et al., 2012; Cao et al., 2012; Liang et al., 2012; Li et al., 2013b). Extra studies found that icariin treatment could significantly induce the activation of ERK, JNK, and p38 kinase, and their respective inhibitors would dramatically attenuate icariin-stimulated osteogenic effects. Ye et al. found that TAZ (the transcriptional coactivator with PDZ-binding motif) depletion could significantly block the promoting proliferation and osteogenic differentiation induced by icariin treatment (Ye et al., 2017). These studies indicated the involvement of Wnt/β-catenin-BMP2, Notch, MAPK, and RhoA-TAZ signalling pathways in the osteogenic effects by icariin (Song et al., 2013; Wu et al., 2015a; Ye et al., 2017). Additionally, the osteogenic differentiation ability of BMSCs from OVX rats would be significantly decreased compared with that in the sham operation group. While icariin treatment could act to protect and increase the osteogenic differentiation and mineralization *via* the estrogen pathway (Luo et al., 2015). Icariin could also protect osteoblasts cell cycle and suppress their apoptosis induced by oxidative stress. There was less production of reactive oxygen species and malondialdehyde, and more superoxide dismutase activity with the treatment of icariin (Liu et al., 2012a). Therefore, icariin could effectively preserve potential osteogenic differentiation of the cells in hypoxic condition, with the increased levels of *RUNX-2*, *OSX*, and *BMP-2* gene expression, and the functions of ALP activity, and mineralized nodules (Liu et al., 2012a).

Icariin not only stimulated osteogenic differentiation but also suppressed the osteoclastogenesis and inhibited bone resorption activity *in vivo*. It was found that icariin could effectively control the proliferation and differentiation of hemopoietic cells which could develop into osteoclasts at the concentration of 10 mM. With the exposure of icariin, the TRAP-positive multinuclear cells appeared to be less. The formed bone resorption pits were inhibited and the osteoclastogenesis-related expressions of *TRAP*, *RANK*, and *CTR* genes were controlled by icariin (Chen et al., 2007a). Huang reported that icariin could suppress the bone resorption functions of osteoclasts *via* the affection on cytosolic free calcium, actin rings, and superoxide generation (Huang et al., 2007). The positive activities of TRAcP, and the activities of osteoclasts formation and bone resorption stimulated by LPS were diminished by icariin. Correspondingly, the synthesis of cyclo-oxygenase type-2, prostaglandin E2, hypoxia-inducible factor-1, and the activation of p38 and JNK were inhibited (Hsieh et al., 2011). Additionally, icariin could inhibit Ti particles-stimulated increase of RNA expressions of the RANKL, CTSK, TRAcP, and MMP9 in RAW264.7 cells. The expressions of IL-1β and TNF-α were increased induced by Ti particles of RAW264.7 cells had also been inhibited (Cui et al., 2014). These experiments indicate the potential inhibitory effects of icariin on the prevention of inflammatory bone loss diseases.

*In vivo* studies with OVX rats, the flavonoids treatment of Epimedium Brevicornum could increase the level of serum osteocalcin and decrease the TRAcP with the comparison to untreated rats. The micro-CT result indicated that the parameters of BMD, BV/TV, Conn.D, and other similar indicators in flavonoids-treated OVX rats were obviously better. The bone histomorphometric parameters of OS/BS, MAR, and BFR/BS were improved. In the mechanical testing, the OVX would induce the reduction of the failure force. However, it was effectively inhibited by flavonoids treatment. While no increase of uterus weight was found during the treatment progress (Zhang et al., 2006; Peng et al., 2009; Liang et al., 2012). The experiments *in vivo* with C57BL/6 mice found that icariin could prevent decreased BMD and bone strength in femur by oestrogen deficiency after ovariectomy surgery (Mok et al., 2010). The ratio of OPG/RANKL expression in tibia has been improved (Mok et al., 2010). In the OVX rat experiment, orally treated rats with icariin at the concentration of 125 mg/kg body weight enhanced the activity of bone mineralization and formation, obtaining higher BMD, biomechanical, and histopathological parameters. And the decreased concentrations of Ca^2+^, P, and E_2_ in the serum were prevented (Nian et al., 2009). In the glucocorticoid-induced osteoporosis (GIOP) model study, icariin significantly attenuated the bone deteriorations, less BMD, hypocalcemia, and hypercalciuria of glucocorticoid positive group. The bone formation level of ALP, calcium, OCN, and fibroblast growth factor-23 in serum were increased. The bone resorption markers of carboxyterminal collagen cross-links, C-terminal telopeptide of type I collagen, and TRAP were reduced (Feng et al., 2013; Zhang et al., 2015). The antiosteoporotic effects by icariin maybe act *via* involvement of the ERK, PI3K/Akt/GSK3b/β-catenin integrated signalling pathways (Feng et al., 2013; Zhang et al., 2015). Liu et al. found that icariin had beneficial effects for osteoporotic rats *via* the inhibition of peroxisome proliferator-activated receptor γ (PPARγ) and Notch2 mRNA expression (Liu et al., 2017a). And Ma et al. found that icariin appears to be a therapeutic drug to manage glucocorticoid-induced bone loss *via* the activation of microRNA-186-mediated suppression on cathepsin K (Ma et al., 2018). Additionally, icariin could significantly reduce particle-induced bone resorption by suppressing osteoclast formation (Shao et al., 2015). Oral administration of icariin improved the abilities of bone formation with higher BMD in the regenerated bone area during the distraction osteogenesis of mandibular, indicating the icariin might be a potential medicine could shorten the course and improve the activity of distraction osteogenesis (Wei R. et al., 2011).

In clinical, a double-blind placebo-controlled clinical trial showed that the flavonoids treatment (containing the compounds of icariin, daidzein, and genistein in Epimedium) possessed the beneficial ability to inhibit the serious bone loss in postmenopausal women. The BMD could be maintained at 12 and 24 months with treatment. However, no significant changes in serum estradiol or uterus tissue were found, indicating the safety to endometrium during the application (Zhang et al., 2007a).

Therefore, being the main ingredient of *E. brevicornum*, icariin could act as a potential useful medicine to affect the imbalance of bone metabolism by increasing osteogenesis and inhibiting bone resorption. More importantly, despite the low number of clinical trials with Chinese medicine compounds, and three kinds of flavonoids in the Epimedium treated group, it has effectively indicated the antiosteoporotic effects by *Epimedium Brevicornum Maxim* clinically. Numerous studies in this review based on osteoporotic animal models, osteoblasts and osteoclasts cells have deeply and consistently confirmed the potential effects and mechanisms by which icariin regulates bone metabolism to treat osteoporosis. Furthermore, high-quality clinical research is needed to test the antiosteoporotic effects by the single compound and to compare their representive effects.

### *Pueraria montana* (Lour.) Merr

The Chinese herb of *Pueraria montana* (Lour.) Merr. (PM, “Ge Gen”) has been famously used for the daily diet and medicine in China and other Asia countries from ancient years. Being a classical and antioxidant agent, it had more recently exhibited benefits for the treatment of angina pectoris and hypertension (Yang et al., 2010b; Tan et al., 2017), neurological health (Gao et al., 2009), blood glucose homeostasis (Prasain et al., 2012), and bone metabolism (Manonai et al., 2008).

Puerarin is an active and famous isoﬂavone compound extracted from the classical Chinese medicine *P. montana*. Puerarin treatment with intragastric administration protected against the decreased levels of BMD and BMC, and the poor structure of femur trabecular bone in ovariectomized rats was improved (Wang et al., 2012a). In the *in vivo* study with orchidectomized (ORX) osteoporotic model, the BMD of the femur was significantly decreased. PM treatment of diet intake effectively decreased the impaired BMD, and the analysis of the femoral metaphysis indicated that PM significantly decreased the levels of BV/TV and trabecular number. And the enhancement of trabecular separation in ORX mice was restored (Wang et al., 2005; Yuan et al., 2016). In the experiment with natural menopausal monkeys, the treatment of 1000 mg/kg body weight of Puerarin powder for 16 months could significantly alleviate the loss of cortical bone. And the bone turnover levels of serum ALP and osteocalcin were decreased (Kittivanichkul et al., 2016). Puerarin 6’’-O-xyloside (PXY), one of the major isoflavones of the *P. montana* had the beneficial effects to improve the levels of calcium, phosphorus, ALP activity, and OPG which had been decreased after OVX surgery in ICR mice serum. The destructive femur osseous tissues of enlarged bone marrow cavity and sparse trabecular bone were alleviated with PXY treatment. Correspondingly, PXY effectively improved the proliferation of osteoblasts *via* the improvement in the expression of OPG/RANKL ratio (Li et al., 2016b).

In the *vitro* study, Puerarin could stimulate and improve the proliferation and differentiation of osteoblast cells (Wang et al., 2013a; Wang et al., 2014). The stimulation of osteoprotegerin and inhibition of RANKL and interleukin-6 production may act *via* the classic estrogen response element (ERE) pathway in MG-63 cells (Wang et al., 2014). And the expression of OPG mRNA was increased by Puerarin in MC3T3-E1 osteoblast cells (Yuan et al., 2016). Puerarin at the dose of 2.5-100 µM would increase the growth of human BMSCs concentration-dependently (Lv et al., 2015). The osteoblastic maturation would be stimulated with the increased ALP activity, as well as the formation of mineralized nodules by Puerarin (Wang et al., 2012a; Lv et al., 2015; Zeng et al., 2018). The signalling pathways of classical ER, MAPK, and Wnt/β-catenin were involved in the osteogenesis and bone formation effects stimulated by Puerarin treatment (Wang et al., 2012a). Lv et al. found that the osteogenesis marker expressions of Runx2, osterix, and osteocalcin were enhanced *via* the increased nitric oxide production and cyclic guanosine monophosphate content in hBMSCs (Lv et al., 2015). And Zeng et al. reported that the expression of transient receptor potential Melastatin 3 (TRPM3) and miR‐204 were decreased and the activation of Runx2 was promoted following puerarin treatment in MC3T3‐E1 osteoblastic cells (Zeng et al., 2018). Additionally, Puerarin opposed the apoptosis of human osteoblast cells induced by cisplatin or in the serum-free condition. The expression of Bcl-xL and Bcl-2 was up-regulated and Bax was decreased *via* the activation of MEK/ERK and PI3K/Akt signalling (Liu et al., 2013; Wang et al., 2013a).

PM could also inhibit the formation of osteoclasts *in vitro*. *Pueraria montana* extract (PME) could dose-dependently inhibit osteoclast differentiation and formation from the precursor cells. Consistently, the expression of osteoclast differentiation markers including *c-Fos* and *NFATc1* genes were downregulated (Park et al., 2017). MAPK activity induced by RANKL had also been effectively inhibited by PME treatment (Park et al., 2017). In the *vitro* experiment with RAW 264.7 cells, PM reduced the formation of TRAP-positive cells induced by the stimulation of RANKL. Correspondingly, the mRNA expression of RANKL was inhibited (Yuan et al., 2016).

These results strongly suggest that *P. montana* could act as both effective promotors of osteogenesis and inhibitor of RANKL-induced osteoclastogenesis, and it appears the isoﬂavon compounds of Puerarin and PXY have the great promotion on osteogenesis ability in the *in vivo* and *in vitro* studies. Even Pueraria Montana may be a potential therapeutic agent for the treatment of bone loss diseases, while the definite extracts of PM to inhibit osteoclastogenesis were still not well known and studied. Further research is necessary to characterize the bioactive compounds of CM which contains anticatabolic or anabolic benefits for the treatment of osteoporosis, and their molecular mechanisms providing the antiosteoporotic effects.

### *Salvia miltiorrhiza* Bunge

*Salvia miltiorrhiza*Bunge (SMB, “Dan Shen”) has been widely and classically used in clinical practice and trial for the treatment and prevention of vascular diseases in liver and heart, as well as commonly used for treating trauma wounds and fractures and correcting blood stasis in TCM for its antioxidant properties (Chen et al., 2017b; Zhang et al., 2017a; Chen et al., 2019b). The application of Salvianolate, Salvianolic acid B on the treatment of osteoporosis has been deeply studied (Guo et al., 2014).

Salvianolate could control the metabolism of bones in glucocorticoid-treated lupus-prone mice. Lupus mice usually have a marked bone loss and deterioration due to an imbalance of bone formation and resorption. Glucocorticoid treatment would deeply restrain their bone formation. After the treatment, Salvianolate increased the trabecular qualities of BV/TV, Conn.D, and Tb.Th, and decreased the SMI number in both the untreated and GC-treated lupus mice. The mechanical parameters of bone ultimate load, yield load, and stiffness in treated lupus mice were significantly improved (Liu et al., 2016). Correspondingtly, the bone resorption maker of serum TRAcP was down-regulated and OPG level was increased. The expression of RANKL, IL-6, ROS, and PPARγ was inhibited, while the Runx2 expression was increased in the mice. These results indicated that Salvianolate treatment significantly affected bone metabolism to inhibit bone loss in lupus mice (Liu et al., 2016). The compound of Salvianolic acid B could prevent glucocorticoid-induced decreased BMD, bone strength, and serious architecture, and could effectively enhance the bone formation rate and the local microcirculation with more capillary dilation (Cui et al., 2012).

There are many compounds in *S. miltiorrhiza* having the pro-osteogenesis abilities including water solution, Salvianic acid A, Salvianolic acid B, Tanshinol, and Tanshinone IIA. The water solution of *Salvia miltiorrhiza* improved bone remodelling by enhancing the gene expression of *ALP*, *OCN*, and *OPG* (Chin et al., 2011). Salvianic acid A protected bone metabolism from serious impairment by the stimulation on osteogenesis and the depression of adipogenesis induced by prednisone (Cui et al., 2009). It was reported that Salvianolic acid B had the potential to stimulate the ALP activity of osteoblastic cells (Liu et al., 2007). It could also protect BMSCs differentiation and increase osteoblast activities *via* the increase of Runx2 mRNA expression even with the exposure of glucocorticoid. The glucocorticoid associated adipogenic differentiation was decreased by the regulation of PPARγ mRNA expression (Cui et al., 2012). In the *vivo* study with rat tibia fracture model, Salvianolic acid B could accelerate the early-stage fracture healing for that the callus growth in the fractured bone was significantly greater in the Salvianolic acid B treated group. And the serum ALP level of the fracture rats was enhanced at weeks 1 and 3 postfracture. These findings indicate that Salvianolic acid B is a potential candidate to treat bone fracture and osteoporosis by the promoting effects on bone formation (He and Shen, 2014). In another experiment with zebrafish *in vivo*, dexamethasone exposure had a series of serious impairment to the bone formation, bone mass, and osteoblast-specific genes. While Tanshinol protectively promoted bone formation and bone mass *via* the inhibition of oxidative stress, and the osteoblast-specific genes expression of *Runx2*, *osteocalcin*, *ALP*, and *osterix* were stimulated (Luo et al., 2016). Additionally, Tanshinone IIA blocked the apoptosis of osteoblasts induced by glucocorticoids *via* the inhibition on the Nox4-derived overexpressed reactive oxygen species activities (Li et al., 2015a). And Tanshinone IIA enhanced the differentiation of C2C12 cells to osteoblasts *via* activating the signalling pathways of p38, BMP2/Smad, and Runx2 (Kim and Kim, 2010). It could also enhance the osteogenic differentiation of human periodontal ligament stem cells *via* enhancing the activation of both ERK and Runx2 (Liu et al., 2019).

In the *in vivo* study, after SMB treatment at the concentration of 5 g/kg for 14 weeks, the unbalanced levels of serum ALP, OPG, TRAcP, and RANKL of OVX rats were attenuated. The decreased BMD and bone strength was inhibited, and the impaired bone microstructures were improved. Moreover, the decreased expression of p‐LRP6, IGF‐1, ALP, and OPG were enhanced. While the increased expression of RANKL and CTSK in the tibias and femurs of OVX rats were effectively inhibited by SMB treatment (Liu et al., 2018). Tanshinone VI, extracted from the root of *S. miltiorrhiza*, which could greatly inhibit osteoclast differentiation and bone resorption by disrupting the formation of actin ring. Tanshinone VI appears to prevent osteoclast differentiation by the downregulation of RANKL expression (Nicolin et al., 2010). Kwak et al. reported that Tanshinone IIA inhibited the osteoclast differentiation from the precursors *via* the down-regulation of RANKL-induced high levels of c-Fos and NFATc1 (Kwak et al., 2006). Additionally, in the natural drug screening experiment, maybe tanshinone 1, cryptotanshinone, and 15,16-dihydrotanshinone I diterpenoids and other unknown compounds had a synergistic effect with tanshinone, possessing the antiosteoclastogenesis effects by reducing the formation and function of TRAP-positive multinuclear osteoclasts (Lee et al., 2005; Kim et al., 2008).

These studies highlight the antiosteoporotic effects of *S. miltiorrhiza in vivo* and *in vitro*. Most of the compounds of *S. miltiorrhiza* including Salvianolate, Salvianic acid A, Salvianolic acid B, Tanshinol, and Tanshinone IIA, and so on, have potential antiosteoporosis effects by promoting bone formation *via* increased expression of osteogenesis-related genes and proteins, and by decreasing bone resorptive osteoclastogenesis through the inhibition of reactive oxygen species activity. Compounds in the research of Kim et al. also have the antiosteoclastogenic effects which are not further studied. More research is needed to provide the evidence of the herb and its potent compounds to target osteoporosis in clinical trials, including their mode of application and mechanisms of action.

## Discussion

In summary, with the increasingly ageing population worldwide, the osteoporotic fracture has become a major health and social issue. The side effects caused by hormone therapy and alendronate antiosteoporotic agents have prompted researchers to study natural therapeutic compounds, which may be effective and safe for the treatment of osteoporosis, and with less adverse side-effects.

**Figure 3:**
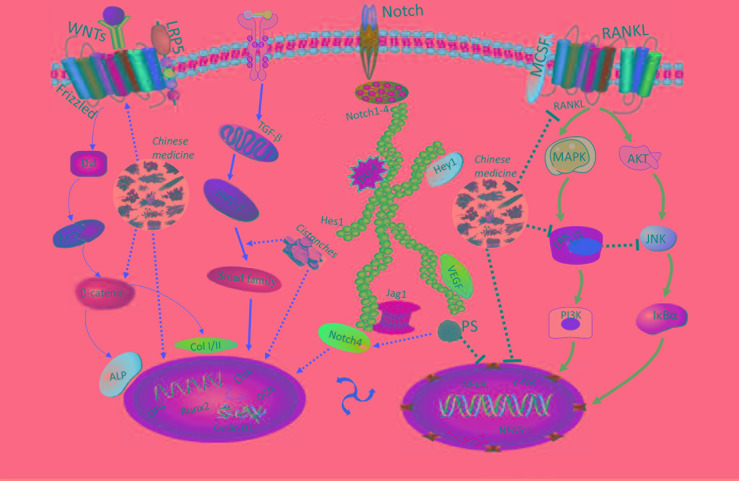
Signalling pathways involved in the anabolic and anticatabolic effects of natural Chinese medicine to treat osteoporosis, including classical Wnt/β-catenin, TGF-β/Smad, BMP2, Notch, RANKL, MAPK, and NFATc1 families.

The pathophysiology of osteoporosis is complicated in terms of occurrence, development, and progression, including much more numerous mechanisms of mechanistic/mammalian target of rapamycin (mTOR), autophagy, and notch involved (Shen et al., 2016; Zanotti et al., 2018; Hiraiwa et al., 2019), except for RANKL, MAPK, Wnt, and Smad signalling pathways discussed above. Natural Chinese medicine may contain compounds that are effective for the treatment of osteoporosis and this review documents current evidence as to their potential bio-pharmacological effects and possible mechanisms of actions. A summary of the *in vivo* and *in vitro* antiosteoporosis effects of the natural herbs reviewed by this article is presented in **Table 1** and **Table 2**, respectively. Natural Chinese medicine appears to promote bone formation activity, including the osteogenesis of MSCs and osteoblasts. Some medicines could protect them from oxidative damage due to ROS activity. Additionally, the bone resorption activity of osteoclasts may be significantly inhibited by certain herbal compounds, thus potentially alleviating the imbalance between bone formation by osteoblasts and bone resorption by osteoclasts. **Figure 3** summarizes the signalling pathways that appear to mediate the antiosteoporotic effects of the natural medicine reviewed by this article.

**Table 1:** Summary of *in vivo* studies for the antiosteoporotic effects of natural Chinese medicine.

TCM	Compound	Animal model	Beneficial effects
*Gynochthodes officinalis* (F.C.How) Razafim. & B.Bremer (syn. *Morinda officinalis* F.C.How)	MO extract	OVX rats	BMC, BMD, serum P, Ca2+ and OPG↑ AKP and TRAP ↓(Li et al., 2009; Li et al., 2014b)
		Disuse OP rats	tibia BMD, histomorphometrical parameters↑ osteoblasts↑ osteoclast ↓ (Seo et al., 2005)
	polysaccharides Monotropein	OVX rats	BMC, BMD, mineral element levels↑ (Zhu et al., 2008; Zhang et al., 2016c)
*Curculigo orchioides*	CO extract	OVX rats	BMC, BMD, and serum OPG↑ serum DPD/Cr, TRAcP ↓(Cao et al., 2008)
		rabbits	bone defects↓ (Wong et al., 2007a)
*Psoralea corylifolia*	PC extract	OVX rats	BMD, ash weight and calcium content↑ (Tsai et al., 2007)
		rachitic rats	serum phosphorus, bone calcification and hyperosteoidosis ↑ (Miura et al., 1996)
	Bavachin, bakuchiol	OVX rats	BMD, trabecular parameters↑ (Lim et al., 2009; Weng et al., 2015)
	Psoralen		BV, Tb. Th, osteocalcin ↑ (Yang et al., 2012)
*Eucommia ulmoides*	EU cortex extract	OVX rats	BMD, biomechanical parameters and microarchitecture↑ (Zhang et al., 2009; Pan et al., 2014; Zhang et al., 2014)
		disused OP rats	
	TL	OVX rats	
	EU leaf extract	OVX rats	BMD, biomechanical parameters, serum OC↑ (Zhang et al., 2012)
	EU seed extract	healthy rats	BMD, microarchitecture↑ (Li et al., 2011)
*Dipsacus inermis*	RD extract	healthy rats	bone density, bone histomorphology↑ (Wong et al., 2007b)
	RDE RTS	OVX rats	BMD, BMC, microstructure, Young’s modulus, serum OC and ALP↑ (Liu et al., 2009; Niu et al., 2012; Liu et al., 2012b; Niu et al., 2015a)
		Disuse OP rats	
*Cibotium barometz*	CB extract	OVX rats	BMD, bone strength, bone metaphysis↑ (Zhao et al., 2011)
Velvet Antler	blood	OVX rats	BMD, IGF-1, testosterone↑ (Yang et al., 2010a)
	VA and blood combination	OVX rats	Microarchitecture, strength, serum ALP↑ (Tseng et al., 2012)
	TVAPL	OVX rats	BWC, BMC, BMD, microarchitecture↑ (Zhang et al., 2013)
*Cistanche deserticola*	CD extract	OVX rats	BMD, BMC, biomechanical parameters↑ (Liang et al., 2011) E_2_, Smad1, Smad5, TGF-β1 and TIEG1↑ (Liang et al., 2013)
	Echinacoside	OVX rats	BMD, microarchitecture and biomechanical parameters↑ (Li et al., 2013a)
*Cuscuta chinensis*	Kaempferol	OVX rats	BMD, Young’s modulus↑(Nowak et al., 2017)
*Cnidium monnieri*	osthole	bone fracture	bone growth, maximum load↑ (Zhang et al., 2017b)
		OVX rats	maximal load↑ (Li et al., 2002)
		heathy rats	peak bone mass, serum OC, micro-architecture, biomechanical parameters↑ (Cheng et al., 2014)
*Epimedium brevicornum*	Icariin flavonoids	OVX rats C57BL/6 mice and GIOP	BMD, serum ALP, OC, micro-architecture, biomechanical parameters↑(Zhang et al., 2006; Peng et al., 2009; Liang et al., 2012) Serum TRACP 5b, CTX↓(Feng et al., 2013; Zhang et al., 2015)
Pueraria montana	Puerarin Puerarin 6’’-O-xyloside	ORX and OVX rats, monkeys	BMD and BMC, serum ALP, OCN↑ (Wang et al., 2005; Kittivanichkul et al., 2016; Yuan et al., 2016)
*Salvia miltiorrhiza*	Salvianolate Salvianolic acid B	Lupus mice GIOP	bone mechanical parameters, RUNX2 expression↑ Serum TRACP, RANKL, IL-6, ROS↓(Cui et al., 2012; Liu et al., 2016)

They could enhance the BMC, BMD, and biomechanical parameters of the bones in osteoporosis model animals. And some of the osteogenesis makers of phosphorus, Ca^2+^, osteocalcin, osteoprotegerin, and ALP in serum would be alleviated during the dynamic metabolism progress of bone formation and resorption.

**Table 2:** Summary of *in vitro* studies for the antiosteoporotic effects of natural Chinese medicine.

TCM	Compound	Cells	Beneficial effects
*Gynochthodes officinalis* (F.C.How) Razafim. & B.Bremer (syn. *Morinda officinalis* F.C.How)	Bajijiasu	OCs	osteoclast formation↓ (Hong et al., 2017a)
	anthraquinone	OBs	proliferation↑ (Wu et al., 2009)
		OCs	differentiation, TRAcP activity↓, apoptosis↑ (Wu et al., 2009; Bao et al., 2011)
	Monotropein	OBs	Proliferation, mineralization↑ (Zhang et al., 2016c)
*Curculigo orchioides*	Curculigoside (CCG)	hAFSCs	osteogenesis, ALP activity, calcium deposition↑ osteoclastogenesis↓ (Liu et al., 2014a)
		BMSCs	Proliferation, differentiation↑ (Shen et al., 2013)
		OBs	oxidative damage↓, proliferation, differentiation↑ (Wang et al., 2012b)
	M2, CCG-A, CCG-B etc.	OBs	proliferation, differentiation↑ (Wang et al., 2017a)
		OCs	TRAcP activity↓ (Jiao et al., 2009; Wang et al., 2013b; Wang et al., 2017c)
*Psoralea corylifolia*	bavachin and bakuchiol	OBs	Proliferation, differentiation, ALP activity↑ (Lim et al., 2009)
	PSO	MSCs	differentiation, ALP activity↑ (Yang et al., 2012)
	Neobavaisoflavone	OBs	differentiation, ALP activity↑ (Don et al., 2012)
	bavachalcone	OCs	osteoclastogenesis, resorption pits↓ (Chai et al., 2018)
*Eucommia ulmoides*	5-HMF	MSCs	osteogenesis Mineralization↑ (Tan et al., 2014)
			adipogenesis↓ (Tan et al., 2014)
	AU, GP, GA, TL	OBs	proliferation↑ (Ha et al., 2003; Zhang et al., 2014)
		OCs	proliferation, differentiation↓(Ha et al., 2003; Zhang et al., 2014)
*Dipsacus inermis*	RDE, RTS	OBs, MSCs	proliferation, differentiation↑ (Liu et al., 2012b; Niu et al., 2015b)
		OCs	osteoclastogenesis ↓ (Niu et al., 2012)
	Asperosaponin VI	OBs	proliferation, differentiation, mineralization↑ (Niu et al., 2011)
	RD extract	MSCs	differentiation↑ (Kim et al., 2011)
*Cibotium barometz*	RW-Cb	OBs	proliferation, differentiation↑ (Xu et al., 2014)
	CB extract	OCs	Differentiation, TRAcP activity↓ (Nguyen et al., 2009)
Velvet Antler	TVAPL	OBs	Proliferation, ↑ (Zhang et al., 2013)
	FA-, NFA-		Proliferation, mineralization↑ (Lee et al., 2011)
	FA	OCs	Differentiation, TRAcP activity↓ (Choi et al., 2013)
	CE-C		Differentiation, actin ring↓, apoptosis↑ (Li et al., 2007)
*Cistanche deserticola*	CD extract	OBs	mineralization↑ (Li et al., 2012b)
	Echinacoside	OBs	proliferation, mineralization, ALP activity↑ (Li et al., 2012a)
	CD extract	OCs	Differentiation, actin ring↓, ROS↓ (Song et al., 2018)
*Cuscuta chinensis*	CC extract	OBs	Proliferation, differentiation mineralization↑ (Yao et al., 2005; Yang et al., 2009)
			ALP activity, collagen synthesis, BMP-2↑ (Yang et al., 2009; Yang et al., 2011)
			oxidative damage↑, apoptosis↓ (Gao et al., 2013)
	Kaempferol, hyperoside	OBs	ALP activity↑ (Yang et al., 2011)
	Campesterol	OCs	osteoclasts activities↓ (Yao et al., 2005)
*Cnidium monnieri*	Osthole	OBs	Proliferation, ALP, mineralization↑(Jia et al., 2016; Zhang et al., 2017b)
	Bergapten	OCs	Osteoclastogenesis and bone resorption↓ (Chen et al., 2019a)
*Epimedium brevicornum*	Icariin, etc.	OBs and MSCs	Proliferation, ALP, mineralization, COL1a2, OSX, RUNX-2, BMP-2/Smad4 Notch2↑(Chen et al., 2007b; Mok et al., 2010; Fan et al., 2011; Cao et al., 2012; Liang et al., 2012)
		OCs	Osteoclastogenesis, bone resorption, RANKL, CTSK, TRAcP and MMP9↓(Chen et al., 2007a)
*Pueraria montana*	Puerarin	OBs, MSCs	proliferation and differentiation, ALP activity↑ (Wang et al., 2013a; Wang et al., 2014)
		OCs	osteoclast differentiation and formation↓(Park et al., 2017)
*Salvia miltiorrhiza*	Salvianic acid A Salvianolic acid B Tanshinol and Tanshinone IIA	OBs,	ALP, OCN, OPG, Runx2↑(Chin et al., 2011; Cui et al., 2009)
		OCs	Osteoclast formation and function, c-Fos and NFATc1↓(Nicolin et al., 2010; Liu et al., 2018)

They have beneficial osteogenetic effects by enhancing the proliferation and differentiation of osteoblasts and bone mesenchymal stem cells, improving the activity of ALP and the formation of mineralized nodules. While the osteoclastogenesis and function of osteoclasts are inhibited.

The natural Chinese medicines in this review are classic and bone-specific medicines. As we know, clinical experiences are very important to Chinese medicine. Chinese medicines were classified into different categories with special functions according to the rich practices and experiences in the clinic and the Chinese medicine theories. Some of them were the classic and bone-specific drugs to treat skeleton fractures and bone loss diseases for their beneficial improvement on bone formation. Most of them have the effects and functions to tonify the “Yang” in traditional Chinese medicine, which has an improvement on bone development and metabolism. “Yang-tonifying” medicines are the most popular and classic kind of natural drugs to treat osteoporosis in Chinese medicine (Ju et al., 2014; Li et al., 2015b). Furthermore, all of them are deeply studied possessing both anabolic and anticatabolic effects. They have potential bone-formation effects by enhancing the proliferation and differentiation of osteoblasts and BMSCs, improving the activity of ALP and mineralization formation. Some of them could protect osteoblasts and BMSCs from apoptosis induced by oxidative stress (Liu et al., 2013). While the osteoclastogenesis and bone-resorption function of osteoclasts were inhibited by the treatment of these medicines (Wang et al., 2014). Interestingly, they have the phytoestrogenic or phytoandrogenic effects which might act as the natural and potential alternatives for hormone replacement treatment or alendronate therapies to significantly inhibit the bone loss and improve skeleton development of osteporotic patients. It has been reported that testosterone played a vital basic and clinical role in the homeostasis of skeletal tissue (Ebeling, 2010). In vivo study indicated that the androgen deficiency would significantly lead to an increase of osteopenia in the aged male rats (Erben et al., 2000). Clinically, testicular malfunction induced by androgen deficiency may cause the osteoporosis in old men with increasing bone resorption (Foresta et al., 1984). Numerous studies indicated that these bone-specific drugs contain phytoandrogens (Edouard et al., 2014), which could act as a natural and potential alternative for testosterone replacement therapy (TRT). They could effectively restore the level of serum testosterone and thus significantly improve the bone health and physical condition of patients (George and Henkel, 2014). Some studies found that the compounds from these classical drugs may also possess phytoestrogenic effects (Jiao et al., 2009; Ma et al., 2011; Zhang et al., 2016b), having a similar structure of estrogen conformation and capabilities to bind with estrogen receptors. Therefore, they may regulate bone remodeling *via* estrogen receptor pathway (Wiseman, 2000). More importantly, the application of these drugs exhibiting phytoandrogen and phytoestrogen effects do not appear to cause obvious or harmful side-effects including cardiovascular disease, prostate cancer, and breast cancer, which might be induced by the long-term and large dosage use of testosterone or estrogen replacement therapy (Wiseman, 2000).

However, the development of osteoporosis is very complex in postmenopausal women, elderly men, glucocorticoid-overuse patients, and other patients with metabolic diseases. The mechanisms of action of natural Chinese medicines effective for the treatment of osteoporosis have not yet been well investigated, thus indicating the need for further studies (Ju et al., 2014). Besides, large dosages or long-term usage warrants caution and certain methodologies should be observed. Further research to isolate and characterize the bioactive antiosteoporotic compounds from the classical and bone-specific drugs is necessary to extensively profile compounds for pharmacological usage, especially their safety, efficacy, and potential chemical interactions with other drugs. Studies to determine the special and targeted cellular and molecular mechanisms of natural Chinese medicine compounds are required to develop their potential application for the treatment of osteoporosis, as an effective, safe alternative to primary therapeutic strategies, or in combination with current primary pharmacological treatments. Additionally, few high-quality clinical studies have documented the antiosteoporosis effects of structure well-known compounds, for example, Epimedium-derived phytoestrogen flavonoids were used to treat and inhibit osteoporosis and bone loss of the postmenopausal women in a clinical trial (Zhang et al., 2007a). There are still some limitations and deficiencies of these clinical drug findings, which are studied together with combined medicines in traditional formulas, due to the potential for unknown interactions between the various drugs and nonspecific compounds in this medicine (Wei H. et al., 2011; Wei R. et al., 2011; Shi et al., 2012). Therefore, more high-quality clinical studies with natural Chinese medicines possessing the anabolic and anticatabolic effects are needed in the future.

## Conclusion

Recent *in vivo* and *in vitro* findings suggest that natural Chinese medicine may provide potential therapeutic benefits for the treatment of osteoporosis. Further research is necessary to ensure the safety, efficacy, and specificity of the compounds in Chinese medicines to develop their therapeutic potential. More high-quality clinical researches with these natural medicines are needed to provide greater evidence for the candidate to beneficial and safer antiosteoporotic application.

## Author Contributions

JH, XL, and ZW contributed equally to this work. JH and XL conceived the idea and wrote the manuscript. ZW, SB, and KC helped modify the language and the revision. ZX collected the literature. JX, DL, and SW helped supervise the research and contribute to the final draft of the paper. We thanked JZ, SC, YH, and JC for the help of this review. All authors reviewed and approved the final manuscript.

## Funding

This work was supported by the National Natural Science Foundation of China (No. 81673992), National Natural Science Foundation of China Youth Fund (No. 81904091) and Fundamental Research Funds for the Central Universities (No. 21619307).

## Conflict of Interest

The authors declare that the research was conducted in the absence of any commercial or financial relationships that could be construed as a potential conflict of interest.

The handling editor declared a shared affiliation, though no other collaboration, with one of the authors, XL.

## Abbreviations

ALP, alkaline phosphatase; BMC, bone mineral content; BMD, bone mineral density; BMP-2, bone morphogenetic protein 2; CTSK, cathepsin K; HRT, hormone replacement therapy; hAFSCs, human amniotic fluid-derived stem cells; IGF-I, insulin-like growth factor-I; NFATc1, nuclear factor of activated T cells cytoplasmic 1; OBs, osteoblasts; OCs, osteoclasts; OC, osteocalcin; OPG, osteoprotegerin; OPN, osteopontin; Osx, osterix; RANKL, receptor activator of NF-κB ligand; ROS, reactive oxygen species; Runx2, runt-related transcription factor 2; TRAcP, tartrate-resistant acid phosphatase.
